# Myelin Oligodendrocyte Glycoprotein (MOG) Antibody Positive Patients in a Multi-Ethnic Canadian Cohort

**DOI:** 10.3389/fneur.2020.525933

**Published:** 2021-01-12

**Authors:** Helen Cross, Farahna Sabiq, Nathalie Ackermans, Andrew Mattar, Shelly Au, Mark Woodhall, Bo Sun, Virginia Devonshire, Robert Carruthers, Ana Luiza Sayao, Virender Bhan, Alice Schabas, Jillian Chan, Marvin Fritzler, Patrick Waters, Anthony Traboulsee

**Affiliations:** ^1^UBC MS/NMO Clinic, University of British Columbia, Vancouver, BC, Canada; ^2^Department of Neurology, Catholic University of Louvain, Louvain-la-Neuve, Belgium; ^3^Autoimmune Neurology Diagnostic Laboratory, University of Oxford, Oxford, United Kingdom; ^4^MitogenDx, University of Calgary, Calgary, AB, Canada

**Keywords:** myelin oligodendrocyte glycoprotein (MOG) antibodies, aquaporin 4 antibodies, multiple sclerosis (MS), demyelination, neuroinflammation, neuromyelitis optica

## Abstract

**Introduction:** Myelin oligodendrocyte glycoprotein (MOG) antibody-associated disease is a recently described central nervous system (CNS) inflammatory disorder with phenotypic overlap with Neuromyelitis Optica Spectrum Disorder (NMOSD). NMOSD seronegative patients, and those with limited forms of the disorder, become suspects for MOG antibody-associated disease. We describe a multi-ethnic population with MOG antibody seropositivity from the University of British Columbia MS/NMO clinic.

**Methods:** AQP4-antibody seronegative patients presenting 2005–2016 with CNS inflammatory disease suspicious for NMOSD, as well as 20 MS controls, were retrospectively tested for MOG-IgG1 antibodies by live cell-based assay at Oxford Autoimmune Neurology Diagnostic Laboratory (UK) and by a commercial fixed cell-based assay at MitogenDx (Calgary, Canada). Additional MOG seropositive cases were identified through routine clinical interaction (2016–2018) using one of these laboratories. Clinical data was reviewed retrospectively.

**Results:** Retrospective testing identified 21 MOG seropositives (14 by live assay only, 3 by fixed assay only and 4 by both) representing 14% of the “NMOSD suspects” cohort. One multiple sclerosis (MS) control serum was MOG seropositive. Twenty additional MOG positive cases were identified prospectively. Of 42 patients (27 female), median disease onset age was 29 years (range 3–62; 9 pediatric cases), 20 (47%) were non-Caucasian, and 3 (7%) had comorbid autoimmune disease. Most common onset phenotypes were optic neuritis (23, 55%; 8 bilateral) and myelitis (9, 21%; 6 longitudinally extensive) Three of the patients in our cohort experienced cortical encephalitis; two presented with seizures. Onset was moderate-severe in 64%, but 74% had good response to initial steroid therapy. Cumulative relapse probability for the MOG positive group at 1 year was 0.428 and at 4 years was 0.628. Most had abnormal brain imaging, including cortical encephalitis and poorly demarcated subcortical and infratentorial lesions. Few “classic MS” lesions were seen. Optic nerve lesions (frequently bilateral) were long and predominantly anterior, but 5 extended to the chiasm. Spinal cord lesions were long and short, with involvement of multiple spinal regions simultaneously, including the conus medullaris.

**Conclusions:** Our MOG seropositive patients display phenotypes similar to previous descriptions, including cortical lesions with seizures and conus medullaris involvement. Many patients relapsed, predominantly in a different CNS location from onset. Serologic data from two different cell-based antibody assays highlight the discrepancies between live and fixed testing for MOG antibodies.

## Introduction

Myelin oligodendrocyte glycoprotein (MOG) antibody-associated disease is a recently described central nervous system (CNS) inflammatory disorder. There are a few large published adult case series (1–5), however the full clinical and radiological spectrum, and optimal management are not yet clear. The majority of published studies are based on Caucasian populations.

MOG antibody-associated disease has similarity to Neuromyelitis Optica Spectrum Disorders (NMOSD) in terms of clinical and imaging phenotypes (6), suggesting that patients within the aquaporin 4 (AQP4) antibody seronegative cohort become suspects for MOG antibody-associated disease. Previously published literature suggests approximately 40% of NMOSD AQP4-negative cohorts are MOG antibody positive (7). Additionally, MOG antibodies can be present in 10–20% of idiopathic atypical demyelinating diseases not meeting full NMOSD criteria (7, 8). MOG antibody-associated disease is however a distinctly different disorder from NMOSD, both immunologically and pathologically (9, 10). This distinction means recognition of these patients is important.

The University of British Columbia (UBC) MS/NMO referral clinic is the largest in British Columbia for CNS inflammatory disorders. NMOSD are known to be more prevalent in non-Caucasian populations. Given the multi-ethnicity of the British Columbian population, this clinic serves a NMOSD cohort of over 200 patients. Whilst primarily an adult clinic, some pediatric cases are also referred.

The primary aim of the study was to describe the population of MOG antibody seropositive patients at the UBC MS/NMO referral clinic, both clinically and radiologically, with comparison to other published MOG antibody-associated disease cohorts, as well as to the rest of our “NMO-suspects” AQP4 negative cohort.

A secondary aim was to systematically examine for autoantibody comorbidity [MOG, AQP4, and N-methyl-D-aspartate receptor subunit 1 (NMDAR) antibodies] within patients with CNS inflammatory disorders.

## Methods

We identified a cohort of MOG antibody patients within our clinic from two sources: retrospectively via batch testing of stored serum samples and prospectively via routine clinical testing. Two different laboratories were utilized for the testing.

We searched our database for AQP4 antibody seronegative patients who were seen at the UBC MS/NMO clinic between 2005 and 2016. We included those who were NMOSD criteria positive (11) or had acute disseminated encephalomyelitis (ADEM) (12), longitudinally extensive or severe transverse myelitis (LETM), severe or recurrent optic neuritis (ON), tumefactive brain lesions, and patients with encephalopathy with white matter lesions and/or cortical lesions, with no clear diagnosis. Patients with neurosarcoidosis, lymphoma, stroke, or vasculitis were excluded. Additionally, we included 20 randomly selected patients with clinically definite multiple sclerosis (CDMS) (13) as controls.

Stored serum samples were tested by live cell-based assay at the Oxford Autoimmune Neurology Diagnostic Laboratory, UK, and on a fixed commercial cell-based assay (Euroimmun AG, Lübeck, Germany) by MitogenDx in Calgary, Canada.

Additional MOG antibody positive cases, in most cases tested only at a single center, were identified (2016–2019) through routine clinical testing at MitogenDx or Oxford Autoimmune Neurology Diagnostic Laboratory. Testing for MOG antibodies was at the discretion of the attending clinician, in most cases being sent due to demyelinating presentations atypical for MS or suggestive of NMOSD.

The systematic testing for AQP4-antibodies and NMDAR antibodies was performed for the retrospective cohort at Oxford Autoimmune Neurology Diagnostic Laboratory via cell-based assay. All prospective cases had AQP4-antibodies tested at MitogenDx via cell-based assay.

Prospective testing of NMDAR antibodies (MitogenDx, via cell-based assay) was not uniformly performed for prospective cases. The timing of serum sampling for MOG antibody testing in relation to clinical disease activity was not standardized. The majority of samples were taken at routine clinic visits, which may or may not have been at the time of a relapse. MOG titers were unfortunately not available, nor were serial test results.

We compared the phenotypic features of our MOG antibody positive patients with the published literature, as well as with the AQP4-antibody and MOG-antibody seronegative patients (from our retrospective cohort) who remained in the idiopathic CNS inflammatory disorders category.

Clinical data pertaining to demographics, disease onset and course, clinical syndromes and response to treatment, was collected by 3 clinicians (HC, NA, and AM) via retrospective chart review of the clinic electronic medical records system (established 2015), as well as provincial health databases for earlier clinical interaction records. Detailed ophthalmic examinations were mostly not documented. Cerebrospinal fluid studies were not performed for many patients and this data was therefore not captured.

Radiological review for the MOG positive patients only was performed by a neuroradiologist (FS), by reviewing the magnetic resonance (MR) imaging available for these patients from the time of first disease presentation, or the first MRI within 5 years of this time. T2 lesions were counted and lesion locations and characteristics were noted. Where possible brain, spinal cord, and orbital imaging was reviewed. Where serial imaging was available, only the first scan was analyzed to maintain consistency.

Ethnicity was captured as Caucasian (European ancestry), Asian (Chinese, Japanese, Korean, Vietnamese or Filipino ancestry), South Asian (Indian Subcontinent), or Other (First Nations or other non-Caucasian ancestry). Clinical severity of disease at onset was classified as mild [VA < 20/100, sensory only (excluding marked neuropathic pain), non-disabling motor], moderate (VA ≥ 20/100, ≤ 20/800, marked neuropathic pain, disabling motor, bladder and bowel involvement) or severe (VA > 200/800, inability to walk, incontinence). Where unable to find specific information, we were guided by clinical impression of severity. Response to initial steroid therapy was graded as good (full recovery or minor residual disability), or poor (minimal change in clinical picture or significant ongoing disability) based on the assessment at the subsequent clinic visit (≥1 month later, but the timing of this was not standardized). Recovery on follow-up was assessed at the patient's last recorded clinic visit, and graded as full/very good (no or minimal disability on clinical impression at last follow-up or EDSS ≤ 2.0), predominantly moderate-severe residual visual disability (VA > 20/100 in worse affected eye), predominantly moderate-severe residual motor disability (motor disability affecting function), predominantly moderate-severe residual bladder, bowel or sexual disability (disability impacting lifestyle), or combination disability when more than one moderate-severe category was present.

No new clinical or radiological data, or blood samples were collected specifically for the study.

Statistical procedures were performed using IBM SPSS Statistics, Version 25.0 (IBM Corp., Armonk, NY). An independent samples *t*-test was used to determine if there were significant differences in numerical variables such as age at onset and duration of follow-up.Chi-square tests and Fisher's exact tests were used to compare the clinical data between the MOG seropositive and the double seronegative groups of patients, with *p* < 0.05 considered to be significant. A Kaplan-Meier analysis was used to determine the cumulative relapse curves and cumulative relapse probabilities. Four patients had to be excluded for this analysis due to lack of specific time data for first relapse. A formal comparison of relapse probability at 1 year between the MOG positive and seronegative group was performed with a *z*-test.

All our clinic patients are offered the option of voluntary participation in research conducted at our center. If they provide written consent, their clinical and imaging information is included in our research database. Additional specific ethics approval for this sub-study was obtained (H19-01146) and it was carried out in compliance with the Helsinki Declaration of 1975 for human studies as revised in 2013.

## Results

**Retrospective Cohort**

We identified a total of 146 patients (4% pediatric, 70% female) who fulfilled our search criteria. Twenty-one of these patients (14% pediatric, 52% female) were found to have MOG antibody positivity on testing: 14 by live assay only, 3 by fixed assay only and 4 by both, representing 14% of patients who were clinically suspicious for NMO and seronegative for AQP4 antibodies. One of the CDMS patients was MOG antibody positive by the live assay test. AQP4 antibodies were detected by live cell-based assay in eight patients previously considered seronegative by a fixed test. Two otherwise seronegative patients and one CDMS patient were found to have NMDAR antibodies by the live test (see Table 1). No patient was found to have dual antibody seropositivity.

**Table 1:** Results of antibody testing in retrospective cohort.

	***n* samples analyzed**	**MOG antibody+**	**AQP4 antibody+**	**NMDAR antibody+**	**Remaining seronegative**
Seronegative “NMO suspects” cohort^*^	146	21	8	2	115
MS controls	20	1	0	1	18

* *Previously seronegative for AQP4 antibodies. MOG = myelin oligodendrocyte glycoprotein. AQP4 = aquaporin four. NMDAR = N-Methyl-D-aspartate receptor. MS = multiple sclerosis*.

**Prospective Cohort**

An additional 20 MOG positive cases were identified through routine clinical practice (19 via fixed assay at Mitogen and 1 via live assay at Oxford). One of these patients was also found to have positive NMDAR antibodies.

Across the two cohorts, the median timing of test serum sample collection in relation to disease onset or last relapse was 1 month (range 0–132) (see Supplementary Table 2).

The clinical features of patients identified by the two different tests are presented in Supplementary Table 1, in comparison to the cohort as a whole. The text below refers to the entire MOG antibody positive cohort.

### MOG Cohort Clinical Data

Of the 42 patients (27 female) with a median disease onset age of 29 years (range 3–62; 9 pediatric cases), 20 (47%) were non-Caucasian (9 Asian, 7 South Asian, 4 other), and 3 (7%) had comorbid autoimmune disease (thyroid disease and psoriasis).

Most common phenotypes at onset were isolated optic neuritis in 23 patients (55%; bilateral in eight) and isolated myelitis in nine (21%; longitudinally extensive in six). Other onset phenotypes included brainstem presentations, combinations of optic neuritis, and myelitis and cerebral syndromes [which included focal deficits due to tumefactive lesions (3), seizures due to cortical lesions (1), or ADEM (2)]. As the majority of our cohort was adult, the proportion of cases with ADEM at onset was low.

Severity of disease at onset was moderate-severe in 64%, but the majority (74%) had good response to initial steroid therapy. Twenty-one percent of patients received other acute treatments in addition to high dose steroids, including plasma exchange, mitoxantrone, and intravenous immunoglobulins.

**Figure 1:**
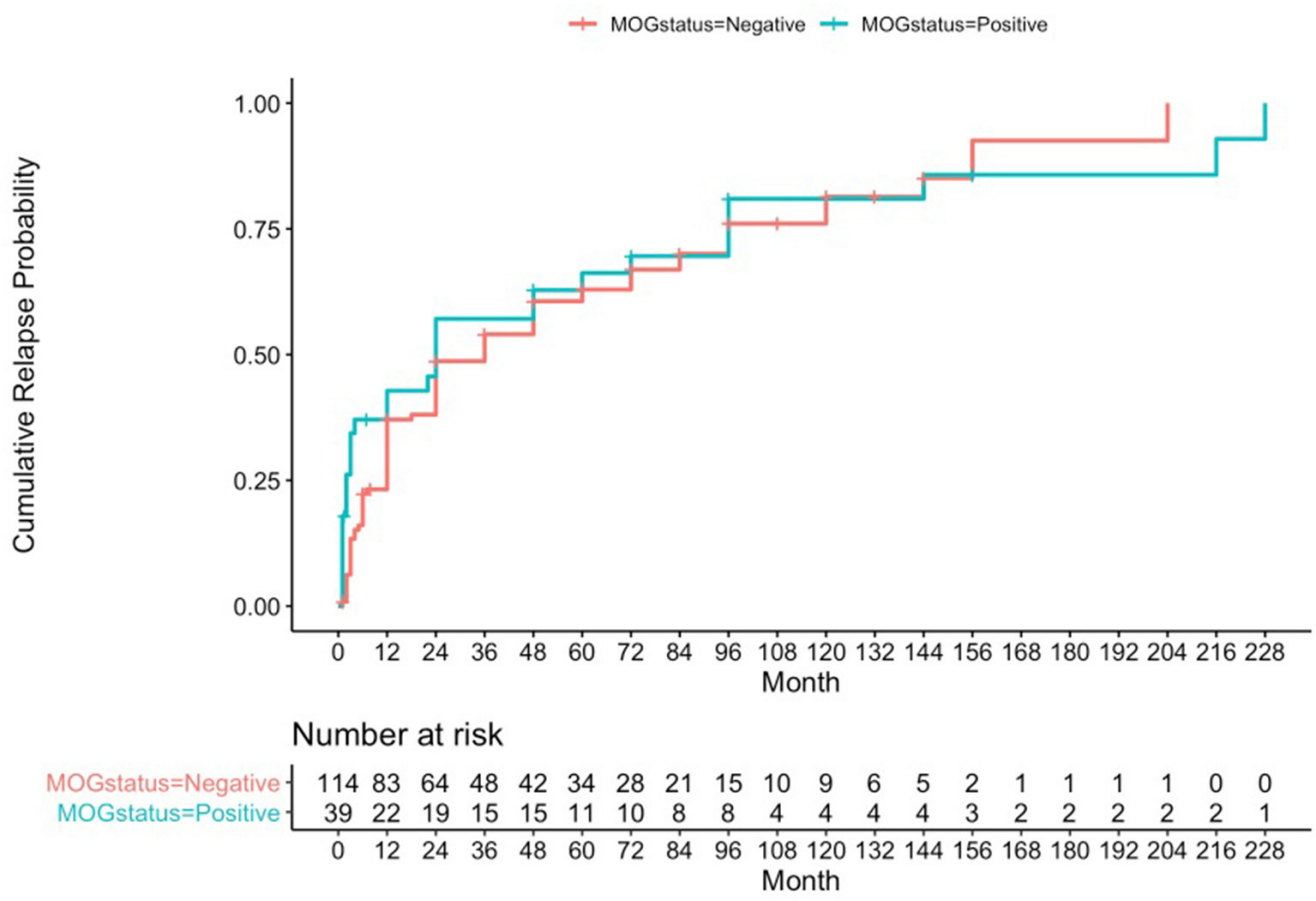
Kaplan-Meier analysis for cumulative relapse probabilities in the MOG-antibody positive and AQP4- and MOG-antibody seronegative groups.

Using a formal Kaplan-Meier assessment, 3 patients who had relapsed had to be excluded due to lack of clarity on exact time to relapse. The cumulative relapse probability for the MOG positive group at 1 year was 0.428 (95% CI 0.244–0.567), at 4 years was 0.628 (95% CI 0.431–0.757), and at 10 years was 0.81 (95% CI 0.602–0.909) (see Figure 1).

Upon examining correlations between onset phenotype and future relapses, it was shown that 68% of patients with isolated optic neuritis and 78% of isolated transverse myelitis relapsed, whilst 100% of those who had presented with cerebral or combination presentations relapsed. Relapses most frequently affected the optic nerves or spinal cord, but the majority experienced different phenotypes on relapse to that experienced at onset (26/34 patients), including brainstem and cerebral or cortical phenotypes. Patients experiencing restricted phenotypes with recurrent disease were most likely to present with optic neuritis (6/7); one patient had pure recurrent transverse myelitis.

No patients had a progressive disease course, although patients with this phenotype are not routinely tested for MOG antibodies at our center.

In total 57% had no or minimal disability at last follow-up. Significant ongoing disability affected predominantly bladder/bowel in 12%, vision in 7%, motor in 2%, and a combination poor outcome was seen in 19%. Chronic steroid-sparing therapies were used in 25/42 patients (59.5%). Azathioprine was the most common first line agent (15/25), but mycophenolate mofetil (MMF) (4/25), and B-cell depleting monoclonal antibody therapies (6/25) were also used. Nine patients required a second line therapy, and one required a third line of treatment. (These constituted mostly MMF and B cell depleting therapies for those not previously using, as well as maintenance IVIG and an interleukin 6 receptor inhibiting monoclonal antibody). Supplementary Tables 2, 3 provide further details on the individual patient presentations.

### Comparison of MOG-antibody Positive and AQP4, MOG-antibody Seronegative Cohorts

In comparison to the MOG-antibody positive patients, the AQP4- and MOG-antibody negative patients showed a higher female predominance 2.5:1 vs. 1.8:1, and a slightly older age at onset (median 39 vs. 29 years).

The seronegative patients were more likely to present with isolated myelitis (57%) at onset than isolated optic neuritis (20%). Onset with neurological deficits in more than one location was more common in the seronegative group (12%) compared to the MOG-antibody positive group (5%). Disability at onset was similarly moderate to severe in both groups, but more patients in the MOG-antibody positive group showed a good response to initial steroid therapy (74 vs. 32%).

Cumulative relapse probabilities in the AQP4- and MOG-antibody seronegative group was 0.371 at 1 year (95% CI 0.274–0.455), 0.606 at 4 years (95% CI 0.498–0.691), and 0.814 at 10 years (95% CI 0.682–0.891) (see Figure 1). A formal *z*-test comparison was performed for estimated relapse probability at 12 months vs. the MOG positive group using their two standard error estimates of 0.04605 and 0.08148, and this was found to be non-significant (*z* = −0.609, 95% CI −0.240 to 0.126, *p*-value = 0.54).

In terms of clinical syndromes experienced at any point during the disease course, MOG-antibody positive patients were more likely to have experienced optic neuritis (74 vs. 40%) or brainstem (38 vs. 21%) presentations than the seronegative group. Myelitis was more common in the seronegative group (80 vs. 52%).

Tumefactive cerebral lesions presenting with the expected focal neurological deficits were common in both groups. Other symptomatic cerebral presentations also occurred, with some difference between the two groups. In the MOG-antibody group, two patients presented with seizures due to cortical lesions. In the seronegative group one patient presented with chorea and another with psychosis.

Both groups had a significant proportion of patients with residual disability affecting function at last follow-up −43% in the MOG-antibody positive group and 51% in the seronegative group (see Table 2).

**Table 2:** Comparison of MOG-antibody positive and AQP4, MOG-antibody seronegative cohorts.

	**MOG-antibody positive**	**AQP4 and MOG antibody negative**	***p*-value**
	***n* = 42**	***n* = 115**	
% female	64	71	0.26
Median age at onset (y; and range)	29 (3–62)	39 (14–78)	**<0.001**
Comorbid autoimmune disease	3 (7%)	18 (15%)	0.17
**Onset location**			
ON	23 (55%) (bilateral in 8/23)	23 (20%) (bilateral in 3/23)	**0.001**
TM	9 (21%) (LETM in 6/9)	66 (57%) (LETM 36/66)	**<0.001**
Cerebral	6 (14%) (3 TL, 2 ADEM, 1 cortical)	5 (4%) (4 TL, 1 ADEM, 0 cortical)	0.069
Brainstem	2 (4.8%)	7 (6.1%)	1
Combination	2 (4.8%) (ON + TM 100%)	14 (12.2%) (ON + TM 8 57%)	0.193
**Clinical syndromes (at any time)**			
ON	31 (73.8%)	46 (40%)	**<0.001**
TM	22 (52%)	92 (80%)	**0.001**
Brainstem	16 (38.1%)	24 (20.9%)	**0.038**
Cerebral	9 (21%) (3 TL, 3 ADEM, 3 cortical)	9 (7.8%) (5 TL, 4 ADEM, 0 cortical)	0.077
Moderate to severe at onset	27 (64.3%)	65 (56.5%)	0.472
Relapse probability at 1 year	0.428	0.371	0.54
Good response to steroids	31 (73.8%)	37 (32.2%)	**<0.001**
**Recovery**			
Full/very good	24 (57.1%)	57 (49.6%)	0.472
Mod-sev bl/b	5 (11.9%)	4 (3.5%)	0.058
Mod-sev visual	3 (7.1%)	11 (9.6%)	0.184
Mod-sev motor	1 (2.4%)	11 (9.6%)	0.184
Combination disability	8 (19%)	28 (24.3%)	0.529
Unclear	1 (2.4%)	4 (3.5%)	1

*y, years; ON, optic neuritis; TM, transverse myelitis; LETM, longitudinally extensive transverse myelitis; TL, tumefactive lesion; ADEM, acute disseminated encephalomyelitis; mod-sev, moderately severe, bl/b, bladder/bowel/sexual dysfunction*.

Chronic maintenance therapies were also used by some patients in the persistently seronegative group (62/115, 54%). Azathioprine was again most common (43/62 patients), but MMF (5) and antiCD20 monoclonal antibodies (5) were also used for some patients, as were traditional MS therapies (10). Second line therapy (predominantly MMF) was used by 22 (19%), and third line (predominantly antiCD20 monoclonal antibodies) by 11 (9.5%).

### Radiological Data

MR imaging was available for 35/42 of the MOG-antibody positive patients (Table 3); 32/35 were performed on 1.5 tesla MRI and three on 3 tesla MRI. Gadolinium contrast was administered in 18/35 cases. The majority of scans (22/35) were performed at the time of first disease presentation.

**Table 3:** MRI results summary: combined MOG cohort.

**MRI available**	**35/42**	**(18 administered Gd)**			
**Brain**	35 MRI				
	9 no lesions				
	26 abnormal	Lesion number	≤3	10 patients	
			4–9	6 patients	
			>9	10 patients	
		Lesion location	Supratentorial	24/26	(Bilateral lesions 18/24: fluffy/poorly demarcated 11/24; 7 of these >2 cm; 5 had CE)
				Subcortical	23
				Cortical	7
				Callosal	6 (nil with diffuse splenium involvement)
				BG	4
				AdjacentV3	1
				No PV lesions	14
				“MS features”	Dawsons Fingers 1, inferior temporal lesions 5, U or S shape juxtacortical 3
			Infratentorial	8 out of 26	All brainstem, 3 with additional cerebellum
				1 diffuse	
				PAG 5 (1 other MB lesion)	
				Pons 7	
				Medulla 4 (2 area postrema)	
				Adjacent V4 6	
				Cerebellar peduncles 6	
**Spine**	27 MRI (5 cervical only, 22 whole cord)				
	15 no lesions				
	12 abnormal	3 STM only			
		3 LTM only			
		6 STM+LTM			
		7 with CM lesions			
		0 with atrophy ≥3 VS			
	Lesions location	Cervical, thoracic, and conus 5			
		Cervical and thoracic 5			
		Thoracic and conus 1			
**Orbits**	14 with dedicated orbital imaging				
	1 no lesions				
	13 with orbital nerve lesions			Long 13	(5 involving chiasm)
				Short 3	
				Bilateral 8	

*Gd, gadolinium; CE, contrast enhancement; BG, basal ganglia; V3, third ventricle; PV, periventricular; PAG, periaqueductal gray; MB, midbrain; V4, fourth ventricle; STM, short transverse myelitis; LTM, long transverse myelitis; VS, vertebral segments; CM, conus medullaris*.

All 35 patients had brain imaging available for review. In nine, no lesions were detected, whilst 26 had abnormal scans. Number of lesions varied widely from ≤3 lesions in 10 patients, four to nine lesions in six patients and more than nine lesions in the remaining 10 patients. Supratentorial lesions were seen in 24/26 patients, bilateral in 18, and “fluffy” or poorly demarcated in 11. Seven of the 24 had lesions larger than two centimeters and five (of these seven) demonstrated contrast enhancement. In the majority of patients (23/26) the lesions were located in the subcortical white matter, but in 14 of these there were no lesions adjacent to the lateral ventricles. Seven patients had cortical lesions. Six patients had lesions in the corpus callosum, but none displayed diffuse splenial involvement. Four patients had lesions in the basal ganglia. One had lesions adjacent to the third ventricle. Whilst five patients had one or two of the components of previously described “classic MS” lesion findings (14) of Dawson's fingers, inferior temporal lobe lesions and S- or U-shaped juxtacortical lesions, no patients had all three characteristics.

8/26 patients had infratentorial brain involvement. In all eight the brainstem was involved, three had additional cerebellar involvement. In terms of location in the brainstem, seven patients had pontine lesions, six cerebellar peduncular lesions and five had lesions in the periaqueductal gray matter. Four patients had medullary lesions (two in the area postrema) and six had lesions adjacent to the fourth ventricle. Only one patient had diffuse brainstem involvement.

Orbital imaging (with minimum coronal T2 views with fat suppression) was available for 14 patients. Thirteen patients showed abnormality in their optic nerves, with bilateral involvement in eight patients. All 13 patients had long lesions, eight of these were exclusively anteriorly situated in the optic nerve, but five extended posteriorly to involve the chiasm.

Spinal cord imaging was available for 27 patients (however five of these had only had cervical cord imaging). 15/27 scans were normal. The 12 abnormal scans showed mixed patterns of involvement: three had only longitudinally extensive lesions (more than or equal to three vertebral segments), three had only short lesions and five had both longitudinally extensive and short lesions. In terms of lesion location, all patients had involvement of more than one spinal region. 7/12 patients had involvement of the conus medullaris. No patients displayed significant cord atrophy.

**Figure 2:**
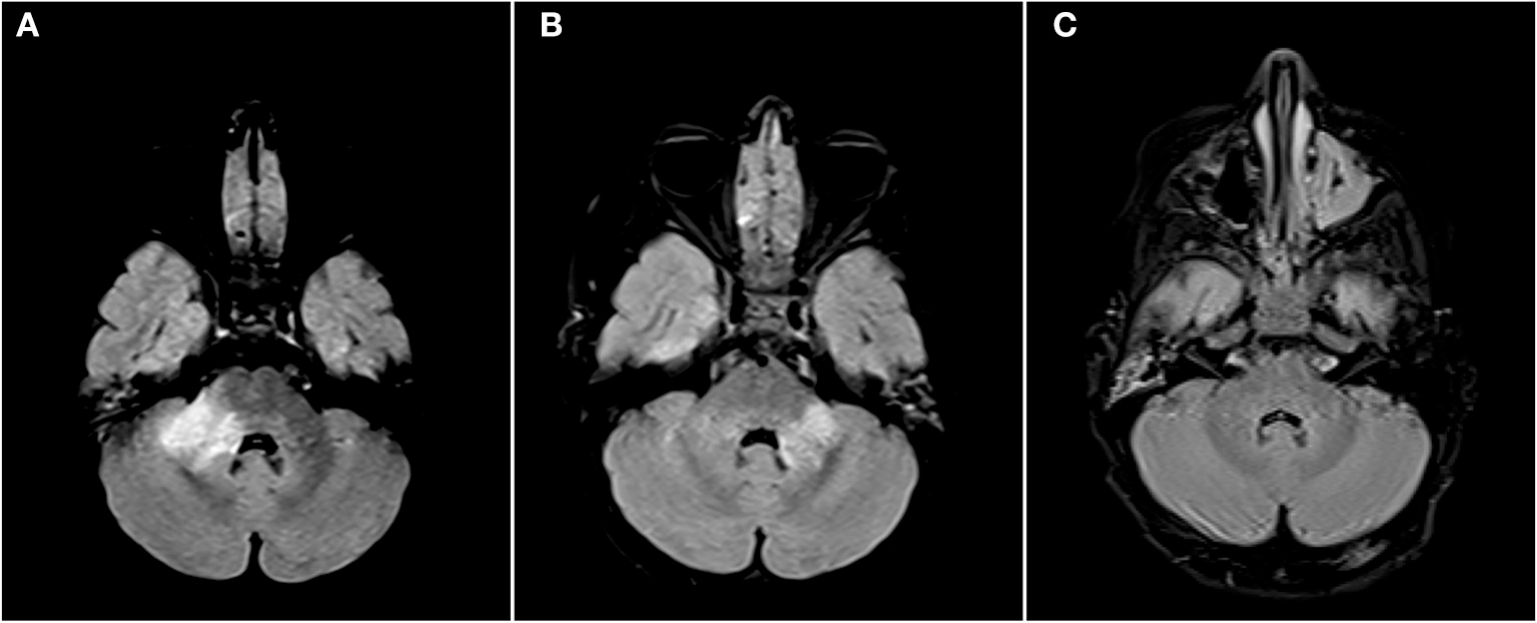
Axial FLAIR images of the brain from the same patient across serial examinations. **(A)** At the time of disease presentation: a FLAIR hyperintense lesion involves the right middle cerebellar peduncle. **(B)** Approximately 2 months later: the lesion in the right middle cerebellar peduncle has nearly completely resolved and there has been interval development of a new lesion in left middle cerebellar peduncle. **(C)** More than 1 year later: The posterior fossa lesions as well as other supratentorial lesions (not shown) have completely resolved.

**Figure 3:**
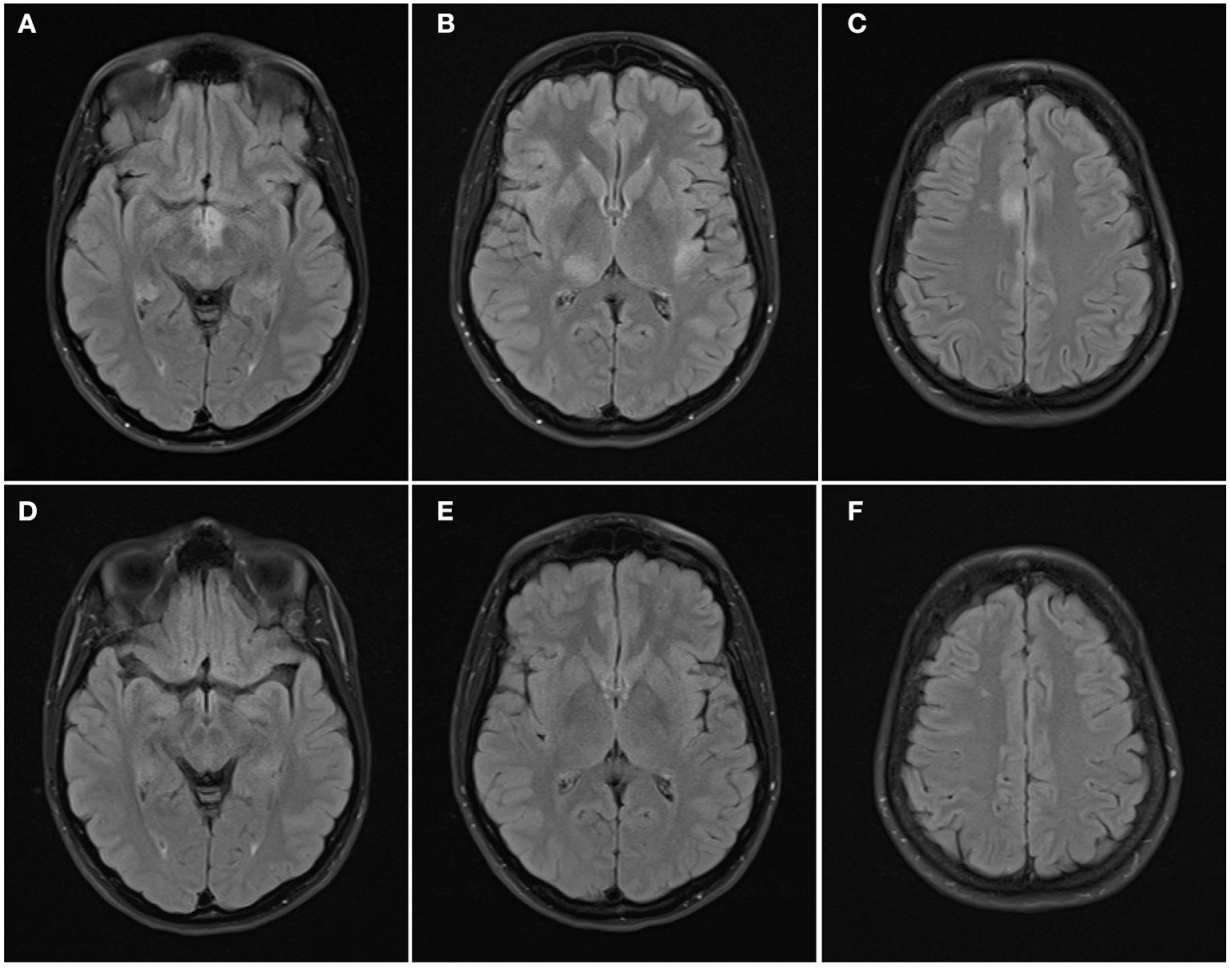
Axial FLAIR images of the brain from the same patient across serial examinations. **(A–C)** At the time of disease presentation: FLAIR hyperintense lesions involve the hypothalamus and optic tracts **(A)**, right thalamus and left insular cortex **(B)**, and parasagittal frontal cortex bilaterally and right centrum semiovale **(C)**. **(D–F)** 5 months later: the lesions have all resolved except for the lesion in the right centrum semiovale.

**Figure 4:**
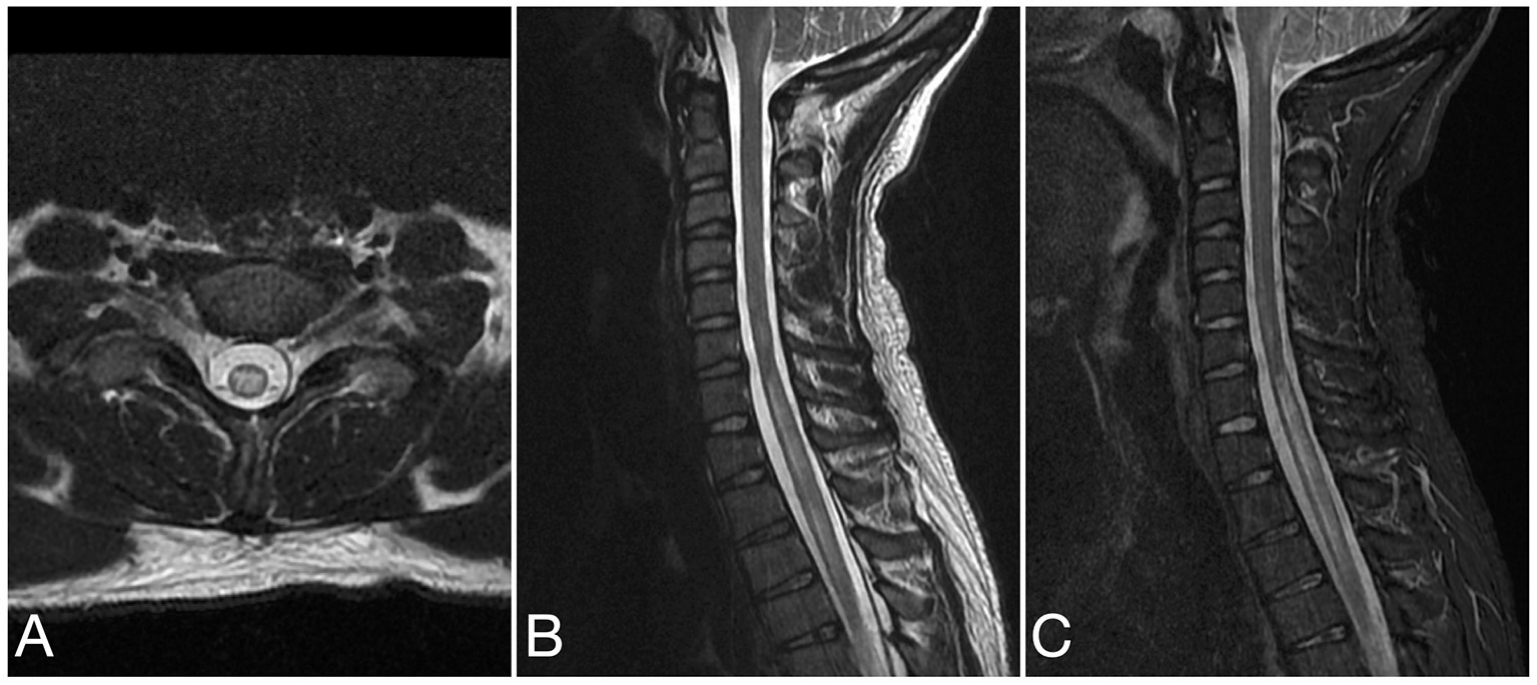
Axial T2 **(A)**, sagittal T2 **(B)**, and sagittal STIR **(C)** images of the cervical spine from the same patient at the time of disease presentation. A longitudinally extensive T2/STIR hyperintense lesion (≥3 contiguous vertebral segments) involve the spinal cord at the cervicothoracic junction.

**Figure 5:**
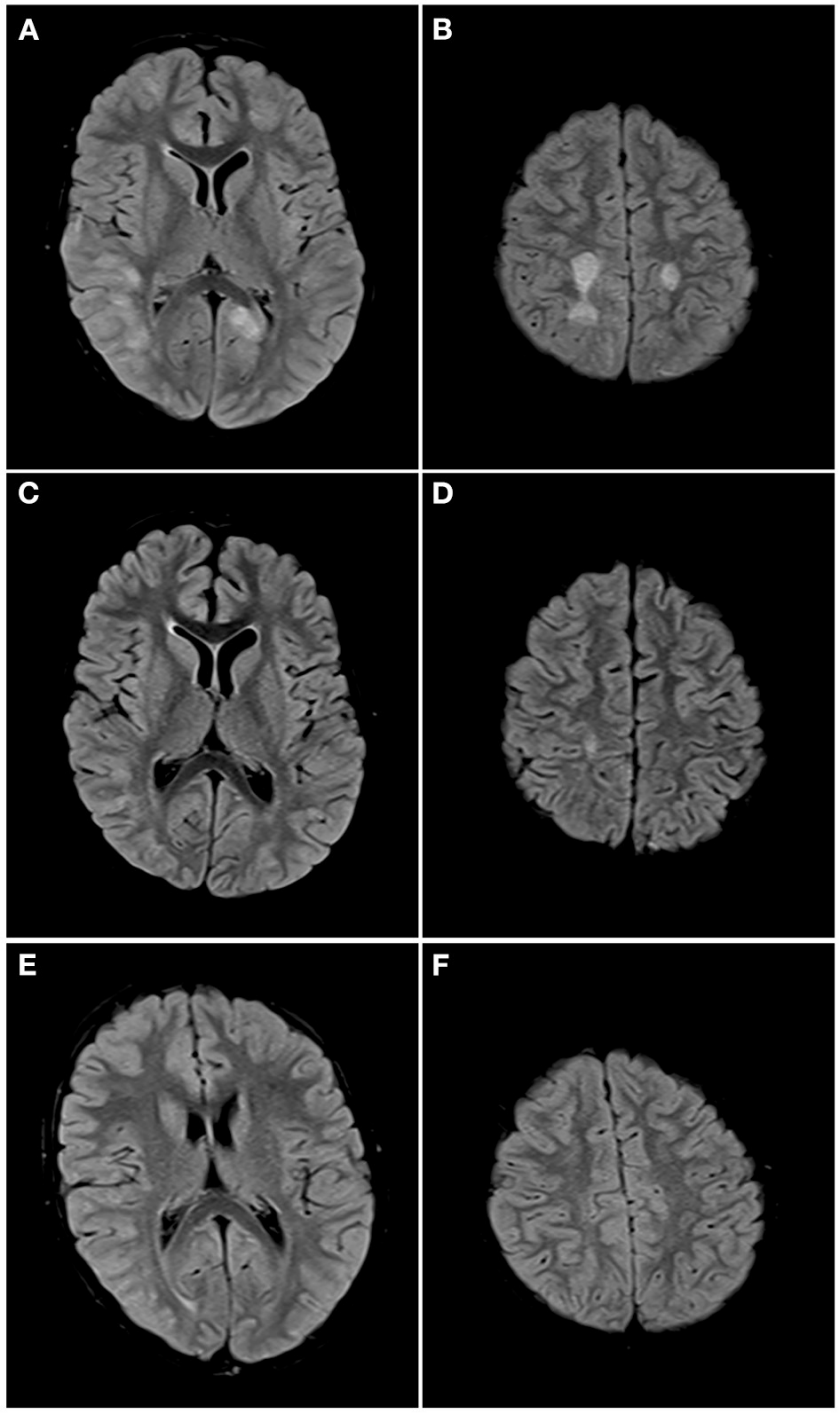
Axial FLAIR images of the brain from the same patient across serial examinations. **(A,B)** At the time of disease presentation: FLAIR hyperintense lesions involve the cortex and subcortical white matter of the right posterior temporal, right lateral occipital and left parasagittal occipital lobes **(A)** and the centrum semiovale bilaterally **(B)**. **(C,D)** Approximately 1 month later: The lesions in both cerebral hemispheres have nearly completely resolved with a small residual lesion in the right centrum semiovale. **(E,F)** One year later: All the lesions have completely resolved.

**Figure 6:**
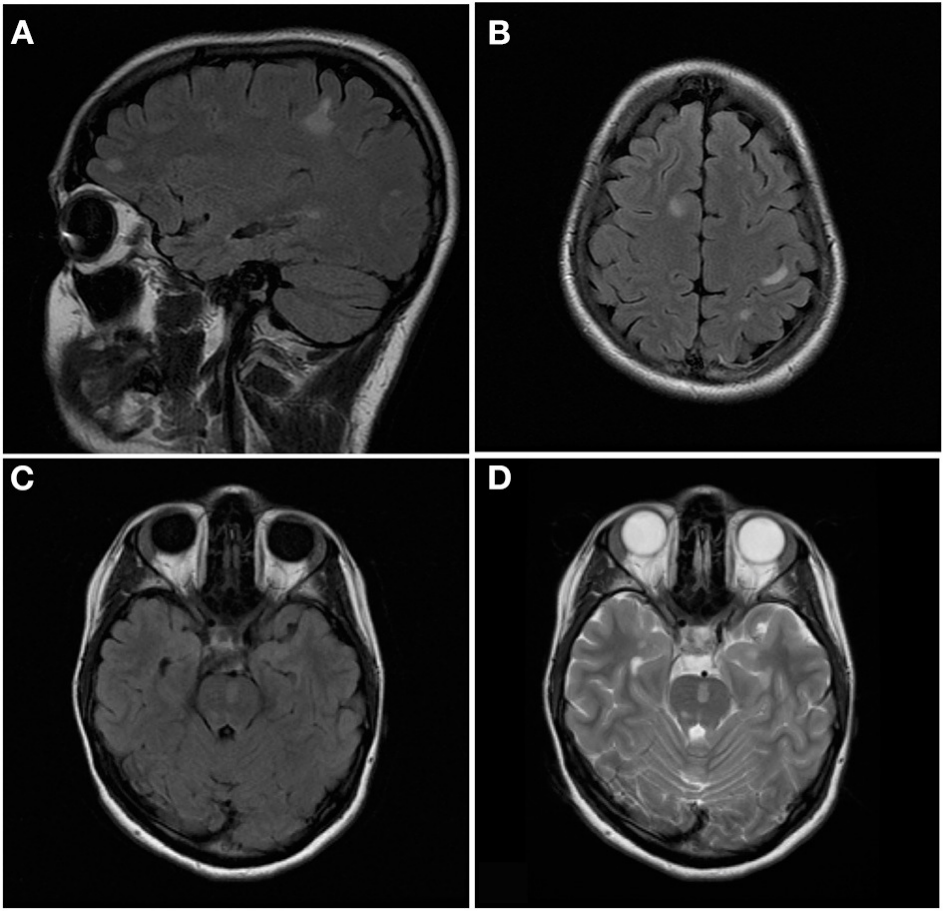
Sagittal FLAIR **(A)**, axial FLAIR **(B,C)**, and axial T2 **(D)** images of the brain from the same patient at the time of disease presentation. **(A,B)** Multiple FLAIR hyperintense lesions involve the juxtacortical and subcortical white matter of both cerebral hemispheres. **(C,D)** Additional T2/FLAIR hyperintense lesions involves the juxtacortical white matter of the left temporal lobe and pons.

**Figure 7:**
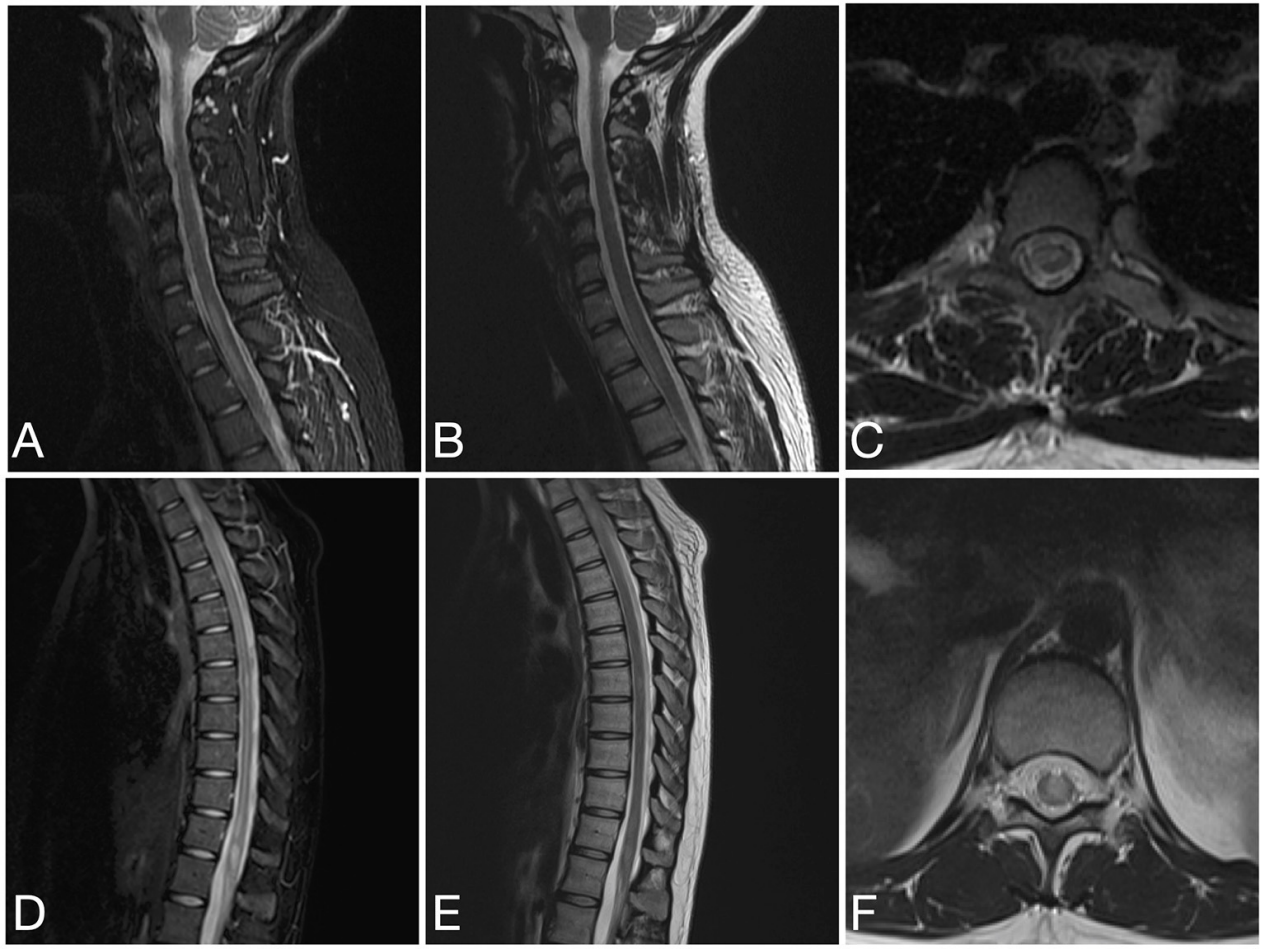
Sagittal STIR **(A)**, sagittal T2 **(B)** and axial T2 **(C)** images of the cervical spine and sagittal STIR **(D)**, sagittal T2 **(E)**, and axial T2 **(F)** images of the thoracic spine from the same patient as in Figure 6 at the time of disease presentation. **(A–C)** A short segment T2/STIR hyperintense lesion (2 contiguous vertebral segments) involve the spinal cord at the cervicothoracic junction. **(D–F)** Other short segment T2/STIR hyperintense lesions involve the thoracic spinal cord and conus medullaris.

**Figure 8:**
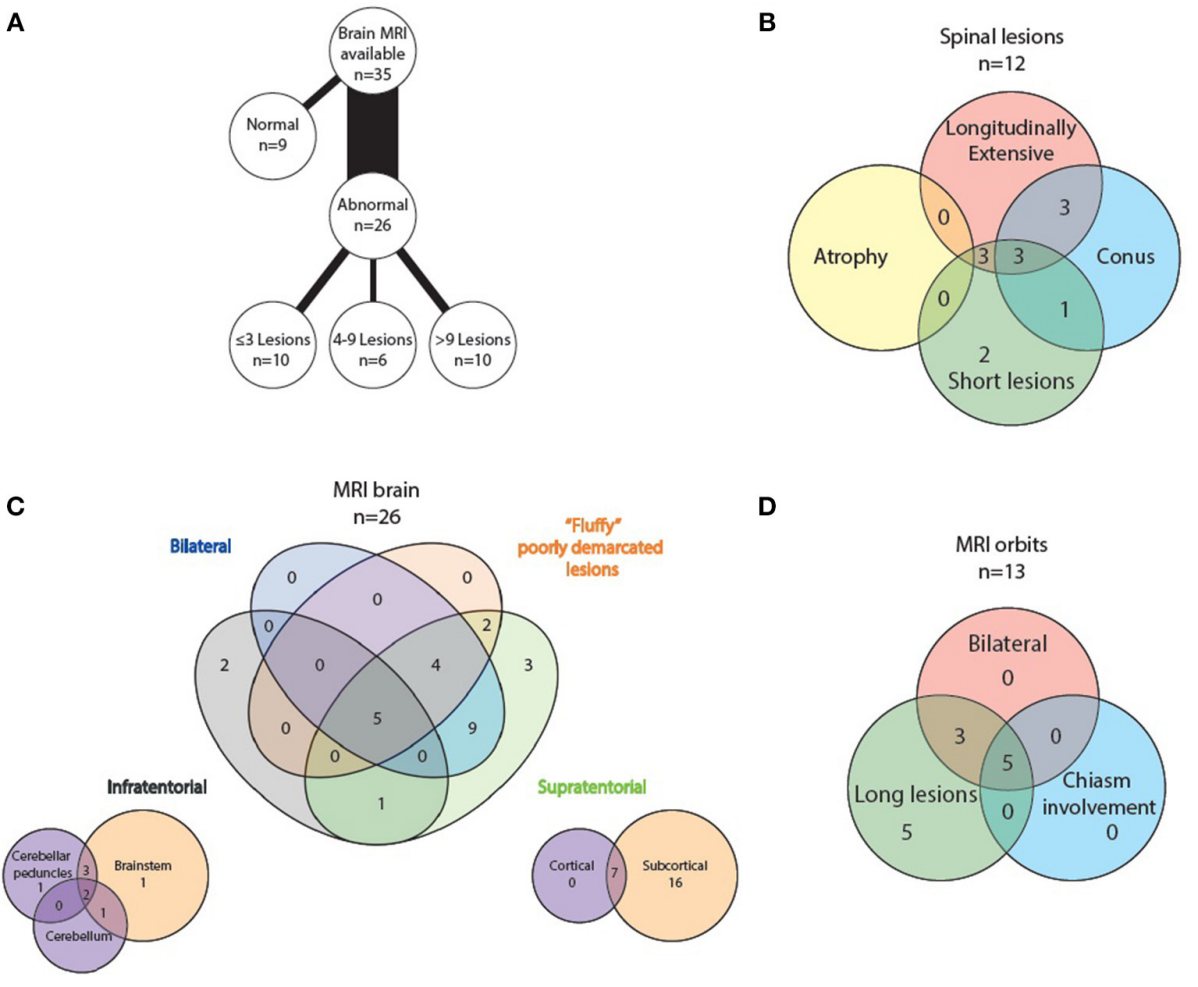
Radiological features of MOG positive MRI scans: **(A)** lesion load on abnormal brain scans, **(B)** spinal lesion characteristics, **(C)** MRI brain topography, and **(D)** optic nerve lesion characteristics.

See attached Figures 2–7 for sample images from our patient cohort, and Table 3 and Figure 8 for a summary of MRI features.

## Discussion

We report a mixed pediatric (9) and adult (33) cohort of 42 MOG antibody positive patients from our multi-ethnic clinic in British Columbia, Canada. Retrospectively we identified 21/146 MOG antibody positives representing a frequency of 14% within the seronegative NMOSD “suspects”/idiopathic atypical demyelinating syndrome cohort which is similar to previous reports (8–20%) (7, 15, 16).

Demographically our patients are similar to other MOG cohorts (1–4, 8) in terms of a balanced gender distribution (64% female vs. 44–68%), onset age (29 vs. 27–37 years) and rate of comorbid autoimmune disorders (7 vs. 7–11%), but our cohort had a higher proportion of non-Caucasian patients (47 vs. 8–27%). Additionally, the onset phenotype was characteristic: 55% with optic neuritis, 21% transverse myelitis (including 7 patients with radiologically confirmed lesions in the conus medullaris, a feature frequently identified in MOG antibody mediated myelitis). With only 9 pediatric patients (median age 7, range 3–14 years) we did not see a high number of ADEM cases, which is typically seen in children under the age of 11 years. This finding was confirmed in a Chinese study examining differences in presentation between adult and pediatric MOG antibody disease (17).

More recently seizures and neuropsychiatric change related to cerebral cortical encephalitic lesions (5, 18–20) have been added to the MOG-antibody clinical phenotype (5, 18–20). Three of the patients in our cohort experienced cortical encephalitis, two of whom presented with seizures. This is not a rare presentation, in fact in a large Chinese series (5), 20.7% of the MOG cohort presented with cortical encephalitis. One of our patients with this presentation on relapse was also found to have NMDAR receptor antibodies. This coexistence of NMDAR and MOG antibodies has been previously described (21, 22). MOG-associated demyelinating episodes can occur simultaneously to NMDAR encephalitis, or can precede or follow (21).

One of our “clinically definite multiple sclerosis” cases was found to be MOG-antibody positive. This patient had presented with recurrent optic neuritis and a mild brainstem relapse, and had minimal MRI lesions. The clinical course of this patient has been atypical for MS, and the attending physician had recently been questioning the diagnosis. MOG antibody disease is felt to be distinct from MS, however as there is not yet a biomarker for MS, we are bound by clinical diagnostic criteria (McDonald criteria) which are limited in their sensitivity and specificity. One caveat of the McDonald criteria is that other potential disorders need to be excluded. A study from Germany (23) examined different groups of MS patients for MOG antibodies and found a rate of 5% MOG-antibody positivity in a group selected for “NMOSD-type presentations” with severe optic neuritis, myelitis and brainstem presentations. The authors suspect that the rate of MOG-antibody positivity in an unselected MS cohort is likely closer to 1%. Overall MOG antibodies are considered a marker of a non-MS disease (23, 24).

Radiologically, MOG antibody disease has some phenotypic overlap with NMOSD but can usually be distinguished from MS (13, 25). Brain imaging can be normal in MOG antibody disease (26, 27), but when lesions are seen they tend to be few, poorly demarcated or tumefactive and most often infratentorial (2, 25, 26). On follow-up imaging, lesions frequently show marked improvement or even resolution (2, 28). Seventy-four percent of our analyzed MRI brain scans were abnormal. The lesion frequency appeared bimodal with 42% (10/24) having <4 lesions and an equal number (10/24) with more than 9 lesions. Infratentorial involvement was relatively less common in our population, however as we only formally examined the first set of MR imaging performed for each patient, this may reflect what we found in our clinical onset phenotypes—that only five percent had brainstem involvement at first clinical presentation. We did not systematically analyse follow-up scans, but lesion resolution was noted in some patients anecdotally which is highlighted with the representative images displayed (see Figures 2–7).

In MOG antibody-associated disease, optic nerve lesions tend to be longer, but more anteriorly situated, sparing the optic chiasm with frequent optic disc edema at presentation (29). Our cohort demonstrated predominantly long lesions, but optic chiasm involvement was seen (5/13), previously reported as rare in this disorder (30, 31).

Spinal cord lesions are most frequently longitudinally extensive, but can also be short (32, 33). This was seen in our cohort with 3/12 abnormal spinal MRIs showing LETM only, 3/12 short lesions only, and both short and long lesions were present in 6/12 scans. Involvement of the conus medullaris is particularly characteristic (32), and was seen in 7/12 of our abnormal spinal MRIs.

Due to the retrospective nature of the study, follow-up times were not standardized so a simple relapse proportion could not be calculated, however a cumulative relapse probability at 10 years was 0.81 (although for this duration *n* = 4). This is a higher proportion of relapsing patients than some other series (1–3), however groups from Germany (33) and Spain (34) report similar high relapse rates of 80 and 78%, particularly optic neuritis relapses. Our cohort is from a referral center for CNS autoimmune disorders and could therefore have a referral bias for more severe or relapsing patients. In addition, due to the selection criteria of the AQP4 seronegative cohort for retrospective testing, it is likely that more severe cases are included in our cohort. Length of initial steroid treatment was not standardized and this data were not available for all patients. It is therefore possible, that early relapses occurred in those with short steroid exposure.

Whilst 57% of patients in our cohort had a good functional outcome at last assessment, 43% were left with significant residual disability after repeated relapses. This is in contrast to earlier reports of MOG-antibody associated disease being relatively benign in comparison to NMOSD, however as our experience with the disease grows, more reports are emerging that indicate that significant (especially visual) disability accrual is in fact occurring in many patients with repeated relapses (4, 33, 35). These data are important for ongoing treatment decisions where lesion resolution is often dramatic and short-term treatment over months is routine.

Our study was not able to incorporate MOG antibody titers or serial test results. We were therefore unable to determine if these factors had influence on disease severity or likelihood of relapse. It has been reported that higher antibody titers at onset may be associated with a more severe disease presentation, but may not be predictive of future relapses (17, 36). Longitudinal persistence of MOG seropositivity may be associated with increased relapse risk (2, 37). The serologic data in this study are from two different cell-based antibody assays, one presenting native human MOG as the substrate (live test) and the other one presenting MOG overexpressed in cells that have been chemically stabilized (fixed test). These tests were discordant. Of the 22 sera retrospectively tested as positive for MOG antibodies, only 4 were positive on both assays, 15 patients were only identified by live testing while 3 were uniquely positive on the fixed test. This highlights the significant discrepancies between live and fixed testing for MOG antibodies which, although not the focus of this study, requires formal investigation (38). In a similar fashion 8/146 serum samples, identified as seronegative AQP4 by stabilized commercial testing (Euroimmun AG), were identified as AQP4 antibody seropositive by live testing on sera that had been stored for many years (range 1–12). These data are not trivial and have important implications for clinical decisions in managing patient care in this severely disabling disease. These data recapitulate multiple other studies (38–40).

## Conclusion

Our multi-ethnic clinic population from British Columbia, Canada display similar demographic and phenotypic features to those previously described. We confirm rarer presenting features or “red flags” suggesting MOG-antibody positivity in patients, such as seizures with cortical lesions and conus medullaris involvement in patients with myelitis. Positive anti-MOG antibodies can rule out MS in patients with an atypical clinical MS disease course. Importantly, many of our MOG-antibody positive patients relapsed and were left with significant disability. International collaborative research efforts could address the clear need for a biomarker to identify patients likely to relapse as well as to establish formal treatment guidelines.

## Data Availability Statement

All datasets generated for this study are included in the article/Supplementary Material.

## Ethics Statement

The studies involving human participants were reviewed and approved by University of British Columbia Clinical Research Ethics Board. Written informed consent to participate in this study was provided by the participants' legal guardian/next of kin.

## Author Contributions

HC drafted and coordinated the manuscript, she was also involved in study design, data collection, and analysis. FS collected the neuroradiology data. NA was involved in study design and initial data collection. AM assisted with data collection. SA performed some of the statistical analysis. MW performed laboratory analyses at Oxford. BS created the venn diagrams. VD, RC, ALS, VB, ASc, and JC contributed patients and assisted with manuscript review. MF coordinated laboratory assessments at MitogenDx and assisted with manuscript review. PW coordinated laboratory assessments at Oxford and assisted with manuscript review. AT was involved in study design, manuscript review, and coordination of the team. All authors contributed to the article and approved the submitted version.

## Conflict of Interest

MF is the director of MitogenDx. He is a paid consultant, has received honoraria or gifts in kind from Inova Diagnostics (San Diego, CA). PW is the director of Oxford Autoimmune Neurology Diagnostic Laboratory. He holds patents for antibody testing and has received consulting honoraria from Biogen Idec, Euroimmun AG, Mereo Biopharma, UBC, and retrogenix. The remaining authors declare that the research was conducted in the absence of any commercial or financial relationships that could be construed as a potential conflict of interest.
